# Linear and nonlinear optical probing of various excitons in 2D inorganic-organic hybrid structures

**DOI:** 10.1038/s41598-020-59457-7

**Published:** 2020-02-13

**Authors:** Mohammad Adnan, Jeremy J. Baumberg, G Vijaya Prakash

**Affiliations:** 1 Nanophotonics Lab, Department of Physics, Indian Institute of Technology Delhi, New Delhi, 110016 India; 2 Nanophotonics Centre, Cavendish Laboratory, University of Cambridge, Cambridge, CB3 0HE United Kingdom

**Keywords:** Materials for optics, Optical materials and structures

## Abstract

Nonlinear optical properties, such as two-(or multi-) photon absorption (2PA), are of special interest for technologically important applications in fast optical switching, *in vivo* imaging and so on. Highly intense infrared ultrashort pulses probe deep into samples and reveal several underlying structural perturbations (inter-layer distortions, intra-layer crumpling) and also provide information about new excited states and their relaxation. Naturally self-assembled inorganic-organic multiple quantum wells (IO-MQWs) show utility from room-temperature exciton emission features (binding energies ~200–250 meV). These Mott type excitons are highly sensitive to the self-assembly process, inorganic network distortions, thickness and inter-layer distortions of these soft two-dimensional (2D) and weak van der Waal layered hybrids. We demonstrate strong room-temperature nonlinear excitation intensity dependent two-photon absorption induced exciton photoluminescence (2PA-PL) from these IO-MQWs, excited by infrared femtosecond laser pulses. Strongly confined excitons show distinctly different one- and two-photon excited photoluminescence energies: from free-excitons (2.41 eV) coupled to the perfectly aligned MQWs and from energy down-shifted excitons (2.33 eV) that originate from the locally crumpled layered architecture. High intensity femtosecond induced PL from one-photon absorption (1PA-PL) suggests saturation of absorption and exciton-exciton annihilation, with typical reduction in PL radiative relaxation times from 270 ps to 190 ps upon increasing excitation intensities. From a wide range of IR excitation tuning, the origin of 2PA-PL excitation is suggested to arise from exciton dark states which extend below the bandgap. Observed two-photon absorption coefficients (β ~75 cm/GW) and two-photon excitation cross-sections (η_2_σ_2_ ~ 110GM), further support the evidence for 2PA excitation origin. Both 1PA- and 2PA-PL spatial mappings over large areas of single crystal platelets demonstrate the co-existence of both free and deep-level crumpled excitons with some traces of defect-induced trap state emission. We conclude that the two-photon absorption induced PL is highly sensitive to the self-assembly process of few to many mono layers, the crystal packing and deep level defects. This study paves a way to tailor the nonlinear properties of many 2D material classes. Our results thus open new avenues for exploring fundamental phenomena and novel optoelectronic applications using layered inorganic-organic and other metal organic frameworks.

## Introduction

High intensity ultrashort laser pulses interact with nanomaterials to produce fascinating parametric and nonparametric nonlinear optical phenomena such as two-photon (or multi-photon) absorption^1^, harmonic generation^2^, coherent anti-Stokes Raman scattering (CARS)^3^, excited state absorption and ultrafast charge carrier dynamics^4,5^. Studies of two- (or multi-) photon absorption in layered materials (such as graphene oxide, MoS_2_ type transition-metal dichalcogenides)^6–11^ have given new insight into novel nonlinear optical device concepts such as optical switching, ultrafast optical generation, etc^12^. Two-photon absorption (2PA) is a well-known third-order nonlinear effect that utilizes two photons (2ћω ≥ E_g_) and takes an electronic transition from ground state to excited state through an intermediate virtual state within the bandgap^7,8,12^. One-photon absorption (1PA, ћω ≥ E_g_) due to its high absorption coefficient results into small penetration depths^11,13^ (∼1/α), therefore conventional photoluminescence (1PA-PL) provides information only from near surface region and is influenced by many unwanted effects such as background emission, photo bleaching, ablation, laser heating effects etc^14^. Infrared excited two-photon induced photoluminescence (2PA-PL) possesses larger penetration depth compared to 1PA–PL^1,4,15^ and has emerged as an important nonlinear optical detection technique, finding applications such as single molecule/nanoparticle detection^16^, fluorescence correlation spectroscopy^17,18^ and multi-photon high contrast bio imaging^19–21^. Such 2PA-PL probed by high intensity ultrashort pulses reveals information from much deeper depths of the sample and several underlying effects^20–22^ (such as lattice defects and layered misalignments) and new excited state relaxations. Further, 2PA-PL has advantage over conventional 1PA-PL in improved spatial confinement, sensitivity and three-dimensional resolution, and specifically in the suppression of unwanted background emission^1,12,15,18,23^.

One of the special class of non-conventional metal-organic frameworks (MOFs) materials in the form of (R-NH_3_)_2_-PbI_4_ (where R is the organic) are crystallographically two-dimensional (2D) materials with alternative stacks of inorganic (PbI_6_ edge shared octahedral extended network) and two organic moieties (R-NH_3_)^+^ inter-digitized within the infinitely 2D extended PbI_6_ network^24–29^. These naturally self-assembled multiple quantum wells (MQWs) show strong room temperature excitonic properties due to quantum confinement and the reduced dielectric screening effects^28–34^. These Mott type excitons (confined within the inorganic bandgap) binding energies are substantially enhanced (~200–250 meV) compared to the parent PbI_2_ (~23 meV) and their optical features have thermal stability even up to 200 °C^28,35–37^. However, such natural self-assembly of layered arrangements are clearly thickness dependent^38,39^, as in the parent PbI_2_. Here, weak van der Waal interlayer forces and organic interlayer conformation play vital roles in the self-assembly process^38^. Therefore, the exciton properties drastically differ from few layers to that of “bulk” as a result of the structural rearrangement of the PbI_6_ network and re-conformation of organic moieties between inorganic sheets^25,39^. It is also demonstrated that for thickness beyond a certain limit, 2D extended PbI_6_ layers become more disordered i.e. Pb-I-Pb in-plane bending becomes more flat, and more strained^40^. As a consequence, the exciton energies are more sensitive to the local environment and the energies are down shifted causing changes in the strain, disorder, and layer structure^25,26^. Since conventional 1PA-PL is restricted to near surface, 2PA-PL is more insightful in probing deep inside the localized excitons, which are affected by inter-layer distortions and intra-layer crumpling^4,13,21^.

In this work we provide an insight of strongly confined excitons from naturally self-assembled MQWs (C_6_H_9_C_2_H_4_NH_3_)_2_PbI_4_ (hereafter CHPI) from linear (one-photon) and nonlinear (two-photon) absorption related photoluminescence (1PA-PL and 2PA-PL). From several linear and nonlinear steady-state and time-resolved PL studies^5,41–46^ utilizing high intensity femtosecond (fs) pulsed lasers, we demonstrate the existence of two distinctly different excitons. These studies further reveal many interesting results such as structurally sensitive excitons, dark exciton non-radiative states and exciton-exciton annihilation processes^41,47,48^. We finally conclude that two-photon induced exciton PL probing is highly sensitive to the self-assembly process restricted by layer arrangement, crystal packing and local environment. Similar results are expected in other 2D materials and our study further paves a way for tailoring nonlinear properties of 2D materials and other MOFs.

## Results

**Figure 1:**
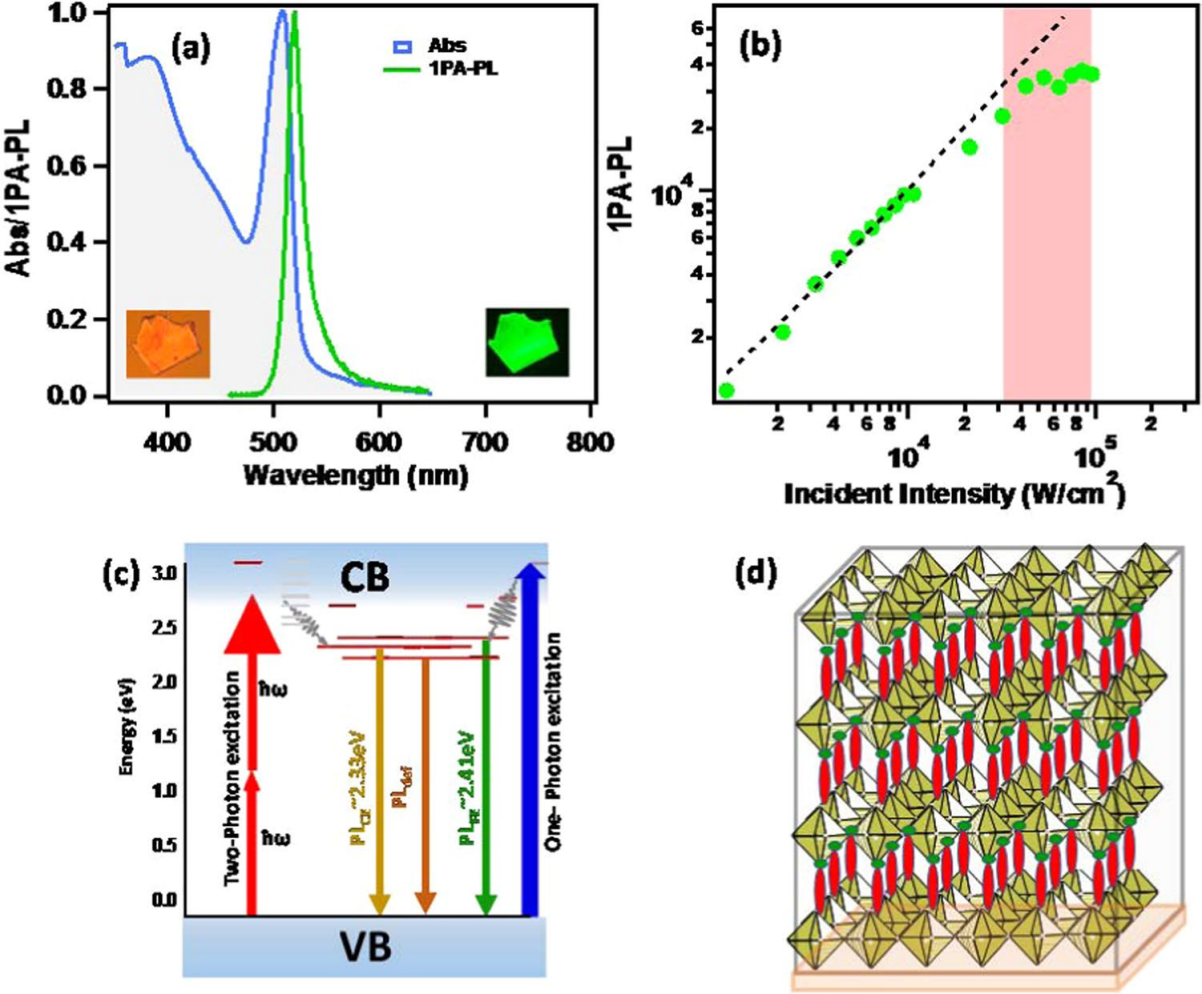
One-photon excited exciton features and possible emission channels of natural MQWs. (**a)** Linear absorption and conventional photoluminescence (1PA-PL) spectra of CHPI thin film (inset shows bright field (left) and PL images (right) of single crystal platelets). **(b)** is laser excitation intensity dependent 1PA-PL peak intensity plot λ_ex_ = 410 nm CW laser. **(c)** Represents schematic energy level diagram showing various possible excitation and de-excitation mechanisms, namely, linear absorption, one-photon (1PA) and two-photon (2PA) absorption induced photoluminescence (PL) processes within inorganic conduction and valance bands and **(d)** Schematic crystal structural packing, where infinitely extended 2D monolayers of [PbI_6_] octahedra are separated by inter-digitized organic moiety layers.

The strong room temperature absorption and one-photon (1PA) excited photoluminescence for one of the typical (R-NH_3_)_2_-MX_4_ type 2D IO hybrid ((C_6_H_9_C_2_H_4_-NH_3_)_2_PbI_4_, hereafter CHPI) thin film is shown in Fig. 1a. The exciton related absorption peak is found to be around 507 nm (2.45 eV) with FWHM of ~20 nm and a band edge at about ~410 nm (~3.0 eV). The conventional optical excitation above the band edge (410 nm) shows corresponding exciton emission at about 515 nm (2.41 eV) (Fig. 1a).

The UV excited conventional PL essentially is one-photon absorption induced (1PA-PL), having ћω ≥ E_g_: the excitation mechanism involves the population of conduction band (band-to-band) of inorganic network and eventually non-radiatively relaxes to the available exciton levels for radiative recombination (Fig. 1c). The excitation intensity dependent peak intensities are plotted in Fig. 1b when excited with 410 nm CW laser. Linearly dependent 1PA-PL peak intensities are limited to low incident laser intensities (in the order of kW/cm^2^) and follow closely the Beer Lambert’s law: ${I}_{PL}\,\propto {I}_{0}{e}^{-{\alpha }_{0}t}$ (α_0_ is one-photon or linear absorption coefficient and *t* is thickness). Continuous wave (CW) excitation causes the PL degradation and ablation (beyond 10^4^kW/cm^2^ excitation intensities), initially due to distortion of layered arrangement and later due to local heating and ablation effects resulting in the modification of PbI_6_ crystal packing^14^.

### Nonlinear one-photon excited photoluminescence

Ultrashort pulse excitation intensities are extremely high enough to produce drastic changes in the excited state population and nonlinear absorption effects as compared to CW excitation. In this study various laser sources are employed namely, (a) 410 nm CW laser and (b) high repetition rate femtosecond laser (690–1040 nm (tunable), 120 fs, 84 MHz: hereafter **fs1**) and (c) low repetition rate femtosecond laser (800 nm, 120 fs, 1KHz: hereafter **fs2**).

**Figure 2:**
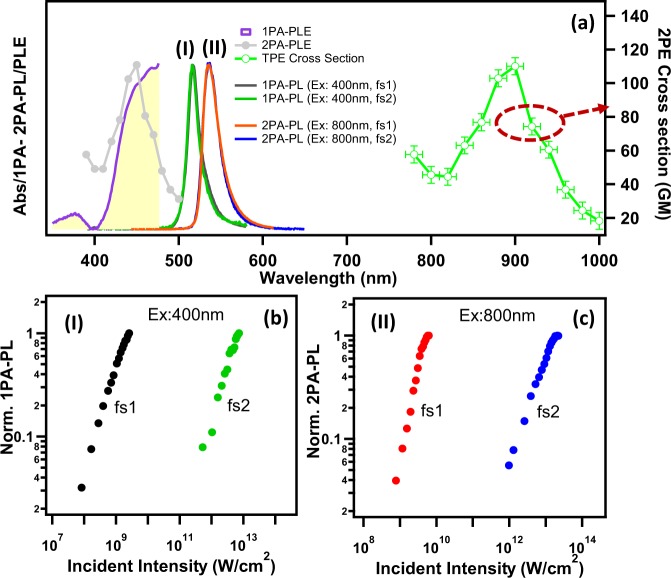
One- and Two-photon excited PL characteristics. (**a**) 1PA-PL spectra of CHPI thin film excited by 400 nm fs1 and fs2 lasers and 2PA-PL spectra excited by 800 nm fs1 and fs2 lasers. The PL excitation spectra of 1PA-PL (1PA-PLE, monitored at 1PA-PL peak, 515 nm), and 2PA-PL (2PA-PLE, monitored at 2PA-PL peak, 532 nm) are also shown here. Right side scale is for the estimated 2PA-PL cross sections (see text). The peak PL intensity vs excitation intensity plots of **(b)** 1PA-PL and **(c)** 2PA-PL when excited by fs1 and fs2 lasers.

Figure 2a depicts the 1PA-PL excited by 400 nm femtosecond lasers fs1 and fs2 (here 400 nm is achieved by frequency doubling the fundamental wavelength 800 nm by using NLO crystal). While the 400 nm fs1 and fs2 laser excited 1PA-PL spectral peak position and width (λ_em_ = 515 nm) are the same as CW excitation, the PL intensity shows multiple nonlinear intensity dependence (Fig. 2b & Supplementary Fig. S6(b)). In the case of femtosecond pulsed laser excitation, the pulse duration is shorter than the excited state lifetimes, therefore the creation of excited states is faster than the radiative decay process. Having such large excitation intensities (in the order of GW/cm^2^), the resultant PL intensities behave nonlinearly and the induced PL intensity (*I*_*PL*_) may be written as ${I}_{PL}\propto {I}_{0}{e}^{-({\alpha }_{0}+\beta {I}_{0})t}$ where I_0_ is the incident laser intensity and β is third-order nonlinear absorption coefficient. The third-order nonlinearity in the present resonant 1PA excitation (>E_g_) mainly arises from nonlinearities such as saturation of absorption, exciton-exciton annihilation and excited state absorption^41,43,49^. The exciton-exciton annihilation is essentially a strong dominant nonradiative phenomena, where two hot excitons strongly interact with each other and one of them recombine non-radiatively by transferring energy to the other exciton which goes to the higher excited state and then relaxes to the lower energy states^41,47^. Due to this, various high-lying energy levels are populated differently and the recombination result in multi-exponential PL decay from various possible exciton sites. Multi-exponential decays and possible exciton sites will be further discussed below.

### Nonlinear two-photon excited photoluminescence

Two-photon excitation (non-resonant excitation, 2ћω ≥ E_g_) is an important nonlinear phenomenon having larger penetration depth (>700 nm) compared to 1PA^1,15^. Two- (or multi-) photon absorption induced PL studies (2PA-PL) are well utilized in many technologically important materials such as GaN QWs, MoS_2_ type 2D materials, graphene oxide and AMX_3_ type 2D&3D IO hybrids^4,6–11^. Such studies are not only helpful in advanced technologies^1,12,17,18^, but also provide fundamental details of excited states– particularly the defect effects on the optical excitons and various exciton energy states below the band gap.

Figure 2a contains 2PA-PL and 1PA-PL spectra excited by 800 nm and 400 nm respectively. The observed 2PA-PL show characteristic narrow exciton emission (FWHM~20 nm) at ~532 nm (2.33 eV), red-shifted by about 17 nm (~70 meV) compared to 1PA-PL. It is also to be noted that both high and low pulse repetition rate excitations (fs1 and fs2) did not show any appreciable change in the 2PA-PL spectral peak position and width. Therefore, it can be speculated that the downshifted 2PA-PL is from the excitons related to the low-lying thickness of interior regions. Having large penetration depth for IR (800 nm) excitation, possibly the 2PA-PL monitors the total thickness/interior region, whereas 1PA-PL comes from near surface region^4,11^. Excitation intensity dependent 2PA-PL (Fig. 2c & Supplementary Fig. S6(c)) show multiple nonlinear slopes > 2, suggesting nonlinear two- (or multi-) photon absorption related effects. Wu *et al*. and Sarmah *et al*. have reported two types of excitons from 1PA and 2PA excitation in mm-sized 3D IO hybrid (CH_3_NH_3_)PbBr_3_ (C1PbBr) single crystals^50,51^. In these bulk crystals, the 1PA excited PL is attributed to surface excitons and the red-shifted 2PA is ascribed to excitons from interior regions which are affected by the bandgap differences due to structural crumpling. In various other reports the thickness dependent red-shift of 1PA-PL in bulk 3D IO hybrid (AMX_3_) single crystals is attributed to either reabsorption mechanisms or band-gap effected excitons^4,52,53^. In general, the exciton features of naturally self-assembled 2D layered system are sensitive to layer stacking and structural distortions, where the weak van der Waal interlayer forces and organic interlayer conformation play vital roles^25,38–40^. In our work, it was also reported that the excitons are sensitive to organic moiety conformation related crumpling of (PbI_4_)^2−^ network when Pb-I-Pb *in-plane* bond angles change from 157.42° to 143.01°, producing up to 190 meV (~39 nm) shift in the exciton energies^54,55^. In another work we have demonstrated that the exciton absorption and 1PA-PL properties of 2D IO hybrid thin films drastically differ between 15–20 layered very thin (<30 nm) film to that of “bulk” (>30 nm) as a result of the structural rearrangement and stiffness of the film^39^. Beyond thicknesses 50–100 nm, the 2D extended PbI_6_ layers become more strained^25,40^. Consequently, the bandgap and exciton energies are sensitive to the local environment due to strain, disorder and layer structure^25,26^. Therefore, it is likely that the PL (at 2.41 eV) from one-photon excitation (ħω > E_g_) is mainly from the top few layers restricted by the penetration depth. On the other hand, the two-photon excited (2ħω ≥ 2E_g_) PL is collected from the entire thickness, therefore the PL mainly reflects the interior region excitons (at 2.33 eV).

The excitation wavelength dependent 2PA-PL spectra are recorded at selected wavelength excitations spanning 780 to 1000 nm using fs1 laser (Supplementary Fig. S1). Corresponding 2PA-PL excitation spectra (2PA-PLE in frequency doubled scale) are plotted in Fig. 2a along with the 1PA-PL excitation spectrum (1PA-PLE). In general, 1PA-PLE closely resembles the linear optical absorption, which contains both band edge and strong exciton absorptions. By contrast, 2PA-PLE represents only those possible nonlinear two-photon excitation energies. Many diverse high-lying exciton states (both radiative (bright) and nonradiative (dark) sites) were previously reported in 2D materials and such excitons are easily dissociated and broadened at room-temperature due to their small binding energies^9,56–58^. Thus, the present 2PA-PLE spectrum further suggests that the 2PA excitation is probing dark exciton states (~2.75–2.61 eV), which can extend below the bandgap energies. During 2PA excitation, the excited electrons relax through free excitons (2.41 eV) to the lowest crumpled exciton state (2.33 eV) (Fig. 1c). To further support the 2PA absorption, we have estimated the third-order optical nonlinear coefficient (two-photon absorption (2PA) coefficient (β)) from open aperture Z-scan^59^ experiment, utilizing 800 nm fs laser (1 KHz, 75 fs). The estimated β value is found to be 75 cm/GW at laser intensities 1.6 × 10^12^ W/cm^2^ (Supplementary Fig. S4). The two-photon absorption coefficient (β) can also be approximated^60^ using material specific values for bandgap (E_g_ = 2.7 eV) and refractive index (n = 2.11) giving estimates close to ~ 2.1 cm/GW at 800 nm. Both these experimental and estimated β values are of the same order of magnitude reported for other organic laser dyes and conventional semiconductors^61,62^. Thickness-dependent two-photon absorption coefficients (β) reported^8^ for one of the (R-NH_3_)_2_-PbI_4_ type hybrid are 211 cm/MW at 800 nm at 0.2 GW/cm^2^ intensity, which is an order of magnitude larger than those of (R-NH_3_)-PbI_3_ type 3D IO hybrid^7^. In general, two-(or multi-) photon absorption coefficients are strongly dependent on morphology (thickness and alignment), linear and nonlinear refractive index, optical bandgap, temperature and laser characteristics (wavelength, pulse duration and repetition rate)^12,62^. The two-photon absorption (2PA) cross sections (η_2_σ_2_) can also be approximated from the following simplified expression^1,17^,

1 $${({\eta }_{2}{\sigma }_{2})}_{CHPI}=\frac{{\langle F(t)\rangle }_{CHPI}}{{\langle F(t)\rangle }_{Rh6G}}\cdot \frac{{n}_{Rh6G}}{{n}_{CHPI}}{({\eta }_{2}{\sigma }_{2})}_{Rh6G}$$

<F(t)> is the integrated PL intensity and *n* is the refractive index. Here Rhodamine 6G (Rh6G) thin film is used as a standard^17^. The refractive index (n) for CHPI is 2.11 and 1.33 for Rh6G. Calculated 2PA cross sections for a fixed laser excitation intensity are plotted against excitation wavelengths in the right axis of Fig. 2a. The maximum 2PA cross section (η_2_σ_2_) value occurs at resonance 2PE wavelength of 900 nm and the value was found to be 110GM [1GM = 10^−50^ cm^4^ s photon^−1^]. These values are higher than the reported values for various fluorescent organic dyes^23^ (0.2 to 95GM, in the wavelength range 690–1050 nm) and metal-organic frameworks^1^ (0.2–7.2GM, at 850 nm).

### One-photon and two-photon excited photoluminescence dynamics

**Figure 3:**
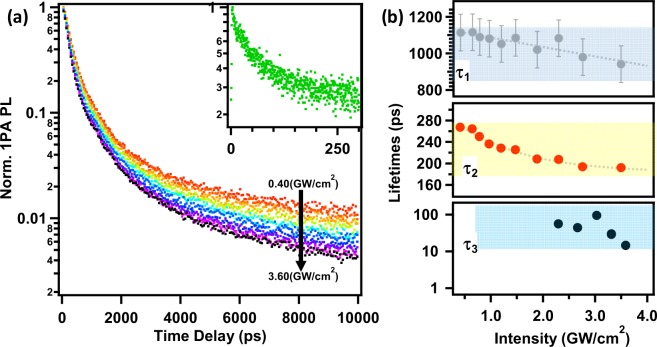
1PA-PL dynamics of various radiative channels. (**a**) Laser excitation intensity dependent time-resolved 1PA-PL dynamics for CHPI thin film and **(b)** fitted lifetimes (*τ*_1_ and *τ*_2_) *vs* excitation intensity. Inset of (**a**) shows upconversion mode data at higher excitation intensity (for I = 2.29 GW/cm^2^) with corresponding lifetimes (*τ*_3_) in (**b**). λ_ex_ = 400 nm (fs1 laser) and monitored at 515 nm.

Now let us understand free (1PA-) and crumpled exciton (2PA-) dynamics. The excitation intensity dependent time-resolved 1PA-PL decay curves using Time Correlated Single Photon Counting (TCSPC, range 0–10 ns, resolution~80 ps) are shown in Fig. 3a. Here the excitation source was fs1 laser (400 nm) and the PL was monitored at 1PA-PL peak maximum, 515 nm. The PL decay curves were fitted by the generic exponential decay function,

2 $$I(t)={I}_{0}+{\sum }_{x=1,2}{A}_{x}\exp (-\,t/{\tau }_{x})$$

where *I*_0_ is a constant, *A* is the amplitude, and *τ* is the PL decay time. The PL decay data are recorded for various intensities from 0.4 GW/cm^2^ to 3.6 GW/cm^2^.

The PL dynamics (Fig. 3a and Supplementary Fig. S7) show bi-exponential behavior with two decay components: a medium lifetime in the region of 190–270 ps (*τ*_2_) as a major component and a longer lifetime component in the region of 800–1200 ps (*τ*_1_) with 10–20% contribution. Figure 3b shows the intensity dependent *τ*_1_ and *τ*_2_ lifetime components. The lifetime *τ*_2_ values are monotonically decreasing with increasing incident intensities (Fig. 3b). These dominant lifetimes (*τ*_2_) are attributed to the free-exciton radiative recombination processes and the values are comparable to those reported for other 2D IO hybrids and transition metal dichalcogenide (2D TMD) semiconductors^5,24,42–47,52,63,64^. The parent PbI_2_ low-temperature (4–180 K) exciton lifetimes are around 500 ps^46^. The thickness dependent carrier lifetimes (185–250 ps for 1–40 μm) in 2D IO hybrid (CH_3_(CH_2_)_3_NH_3_)_2_PbI_4_ have also been recently reported^52^. Gan *et al*. reported a wide range of temperature dependent exciton PL lifetimes (from 300 to 2000 ps) for similar type of 2D layered IO hybrids with picosecond pulse laser excitation^24^. Those large range of decay times were explained based on localized exciton transformation from 0D to 1D like, which is strongly dependent on crystal packing of face-sharing PbI_6_ octahedra^24^. As discussed in previous sections, the PL intensities of 1PA-PL excited by high pump intensities of fs1 and fs2 (Fig. 2b) are strongly influenced by the third-order nonlinearities such as saturation of absorption and exciton-exciton annihilation. However, such nonlinearities can only be expected at high excitation intensities^43^. Delport *et al*. reported PL decay dynamics in (PEA)_2_(MA)_n−1_Pb_n_I_3n+1_ systems using 98 ps laser pulses with laser fluences upto 400 µJ/cm^2^ (upto intensities 4 MW/cm^2^)^48^. In their study the dominant recombination path at high laser excitation fluence is attributed to the exciton−exciton annihilation process and the lower fluence non-exponential decay to defect assisted recombination process. Surrente *et al*. attributed the monotonic decrease of PL lifetimes (laser fluences 0.3 to 1000 µJ/cm^2^, with 300 fs pulses) of 2D black phosphorus to the exciton-exciton annihilation process^41^. Similarly, Sun *et al*. demonstrated the exciton-exciton annihilation nonlinearity in monolayer MoS_2_ from ultrafast transient absorption measurements, utilizing laser fluences from 8 to 46 µJ/cm^2^ (with 300 fs pulses)^47^. Thus, the monotonic decrease of 1PA-PL lifetimes (from 270 ps to 190 ps) with high excitation intensities (from 0.4 to 3.6 GW/cm^2^) in the present study can be ascribed to the dominant effect of exciton-exciton annihilation. The longer lifetime component (*τ*_1_) values are consistently similar throughout all intensities with relatively lower amplitude (10–20%). These lifetimes could be attributed to the bulk crystal domain defects, created by a broad energy distribution of localized states involved in the radiative recombination processes^6,25^. At higher laser intensities, a very shorter lifetime component (*τ*_3_, ranging in between 20–100 ps) is also observed, during short scale decay scans (upconversion mode) (Fig. 3a inset). Though the contribution is less than 10% in overall decay process, these *τ*_3_ decay components may be due to exciton carrier trapping via defect states such as surface defects^41,42,44,45,65^. Surface defect related short lifetimes in other 2D materials such as monolayer black phosphorene(10–70 ps) and MX_2_ type TMDs(1 to 10 ps) were reported recently^41,42,45^. However, these τ_3_ values could be limited by instrumental function and excitation pulse width, therefore further experiments are needed for any confirmation.

The transient dynamics of 2PA-PL as a function of excitation intensity and excitation wavelengths are reported in Supplementary Fig. S2. The 2PA-PL decay kinetics show double exponential behavior with the dominant (>80%) lifetimes ranging between 200–250 ps. These values are close to the 1PA-PL exciton lifetimes reported previously (Fig. 3b). This confirms that the free excitons (1PA-PL) are from those excitons in near-surface perfectly aligned layers, whereas the crumpled excitons (observed in 2PA-PL) are from deeper layers. At these deepest thicknesses, the 2D extended PbI_6_ layers are disordered i.e. Pb-I-Pb in-plane bending becomes flatter and more strained, thus down-shifted exciton energies are expected^39,40^. While both free (1PA) and crumpled (2PA) excitons have similar PL dynamics, they are thus located at two different energies. Further, the excitation intensity dependent exciton 2PA-PL lifetimes are nearly constant until the damage threshold (Supplementary Fig. S2), suggesting that exciton-exciton annihilation plays no role. In all the 2PA-PL dynamics a defect-related longer PL component (800–1400 ps) is also observed with low amplitude (5–10%), which may reflect their non-radiative lifetime which is shorter than for many inorganic-semiconductor localized excitons.

### One and two-photon excited photoluminescence imaging and microscopy

**Figure 4:**
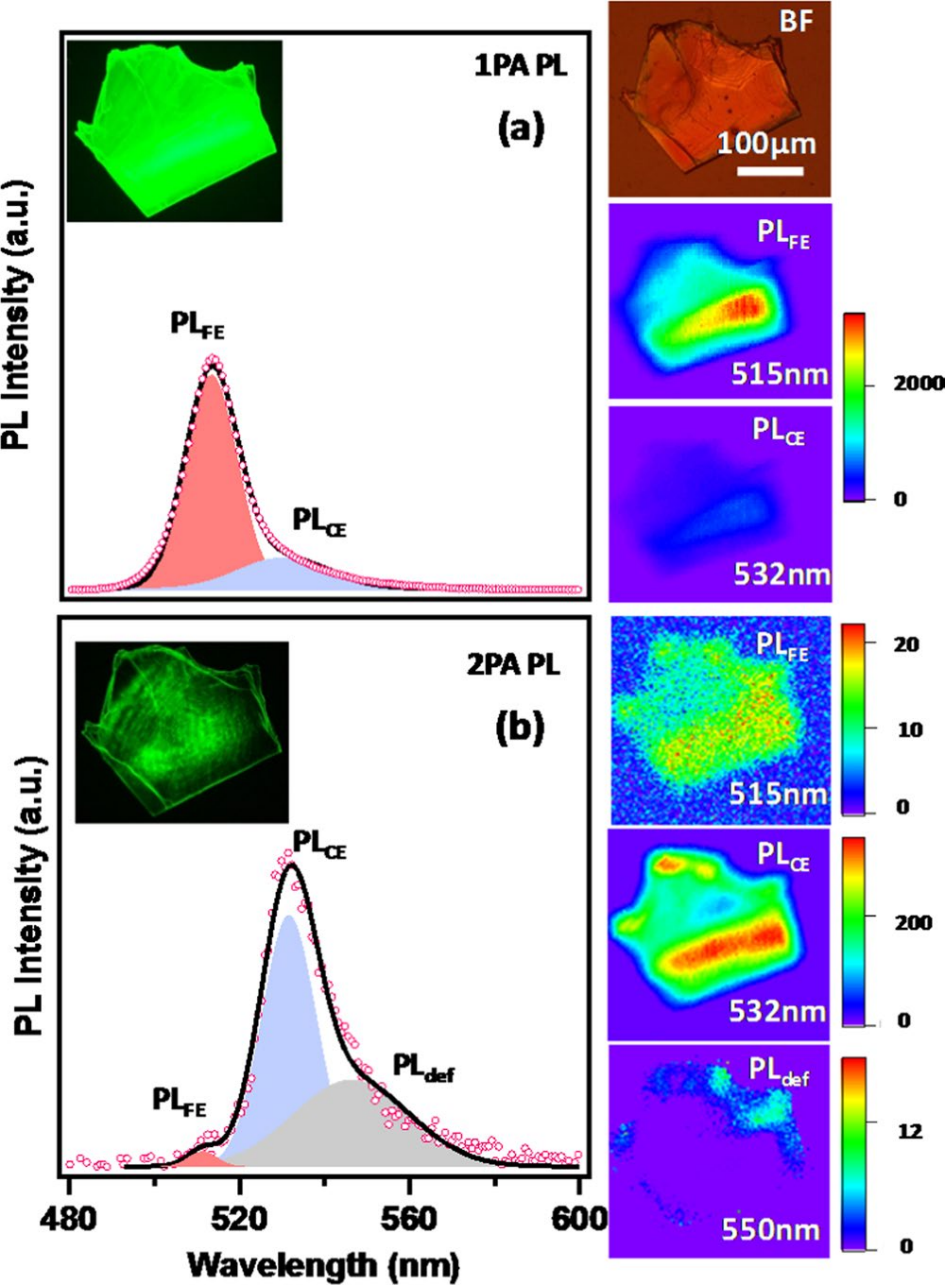
One- and two-photon PL imaging and microscopy of single crystal. (**a)** 1PA-PL and (**b)** 2PA-PL spectra of CHPI single crystal platelet. The spectra are deconvoluted into free-exciton (PL_FE_), crumpled exciton (PL_CE_) and defect induced emission (PL_def_). The respective peak maxima intensity spatial mappings of crystals are shown in the right hand side, along with their relative intensity scales. Respective PL images are shown as inset of **(a**,**b)** respectively. The excitation wavelengths for 1PA-PL and 2PA-PL are 400 and 800 nm from fs1 laser respectively.

Finally, let us focus on co-existence of emissions from (a) high-lying free-exciton, 1PA-PL (PL_FE_) and (b) energy downshifted crumpled excitons 2PA-PL (PL_CE_). 1PA-PL and 2PA-PL spatial spectral mappings (~7 µm spatial resolution) over a CHPI single crystal flakes (300 × 300 µm^2^ area, thickness 100–150 nm) were performed by using 400 nm and 800 nm (fs1 laser) excitation, utilizing a modified confocal microscope (Supplementary Fig. S8). Individual spectra of both 1PA-PL and 2PA-PL are deconvoluted for PL_FE_ and PL_CE_ components and corresponding peak maxima spatial maps are shown in respective Fig. 4a,b (and Supplementary Fig. S9). The single crystal 1PA-PL and 2PA-PL are similar to the spectra obtained in thin film form (Fig. 2a). During 400 nm excitation, the PL_FE_ (515 nm) is dominant emission throughout the crystal area and an order of magnitude lower intensity emission of PL_CE_ (532 nm) is also seen as a shoulder (Fig. 4a). In contrast, in 2PA-PL spectral mapping the PL_CE_ is dominating, whereas PL_FE_ appearance is almost one order of magnitude lower (Fig. 4b). These results are consistent with the previous discussion that one-photon excitation probes free excitons (PL_FE_) those are from top few nearly perfectly aligned layers. Whereas two-photon excitation probes overall crystal (due to larger penetration depth) and the observed PL is dominated by crumpled excitons (PL_CE_) originated from the low-lying thickness distorted (crumpled) (PbI_4_)^2−^ network^54,55^. Some traces of defect induced broad emission (PL_def_ ~550 nm) is also observed at the crystal edges.

## Conclusions

Here we have successfully employed high intensity infrared ultrashort laser pulses to probe into much deeper depths of naturally self-assembled systems. The linear and nonlinear one-photon and two-photon absorption induced photoluminescence (1PA-PL and 2PA-PL) reveal several underlying structural perturbations (such as inter-layer distortions and intra-layer crumpling) and also information about new excited states and their dynamic behavior. Naturally self-assembled inorganic-organic hybrid multiple quantum wells (C_6_H_9_C_2_H_4_NH_3_)_2_PbI_4_ show room-temperature Mott-type exciton emission features (binding energies ~200–250 meV). These excitons are highly sensitive to the local environment and are distinctly different from the UV excited exciton energies to that of two-photon excited excitons. A clear down-shifted 2PA-PL energy (532 nm, 2.33 eV) compared to the 1PA-PL (515 nm, 2.41 eV) suggests the existence of two types of excitons: free excitons originating from perfectly aligned MQWs and crumpled excitons from the distorted 2D layers. Two-photon PL excitation spectra (2PA-PLE) further reveals the fact that the nonlinear excitation invokes dark exciton states, extended below the bandgap. The 1PA-PL excitation intensity dependence and PL dynamics confirm the third order related nonlinear behavior such as saturation of absorption and exciton-exciton annihilation processes. The origin of 2PA excitation is further evidenced by the experimental two-photon absorption coefficient (β = 75 cm/GW at 800 nm) and the estimated two-photon excitation cross-sections (η_2_σ_2_ = 110GM). While both free (1PA) and crumpled (2PA) excitons are located at two different energies, both have similar PL dynamics. The monotonic decrease of 1PA-PL lifetimes with high excitation intensities is attributed to the dominant effect of exciton-exciton annihilation nonlinear processes. Finally, the large area spectral spatial mapping and imaging of 1PA- and 2PA-PL of single crystal platelets clearly demonstrates the co-existence of free excitons and crumpled excitons. Such high intense linear and nonlinear probing of these naturally self-assembled 2D materials reveal many interesting information related to excitons those are highly sensitive to layered arrangement, crystal packing and local environment. Similar is also expected in other 2D covalent and van der Waal materials and the present study further paves a way for the tailored nonlinear properties of 2D materials.

## Methods

### Sample preparation

Synthesis of 2-(1-Cyclohexenyl) ethylammonium tetra iodo plumbate ((C_6_H_9_C_2_H_4_NH_3_)_2_PbI_4_, CHPI) is carried out from the method described elsewhere^25,40^. The harvested single crystals are dissolved in solvents such as Tetrahydrofuran (THF) and dimethyl formamide (DMF). The solutions are spin-coated on glass substrate to obtain films of thickness about 120–140 nm^25^. The single crystal platelets are directly used for imaging and spectral mapping.

### Optical characterization

One- and two-photon absorption induced photoluminescence (1PA-PL and 2PA-PL) for thin films were performed utilizing high intensity femtosecond lasers. The sources are (a) **fs1**: Ti:sapphire mode-locked oscillator (Spectra-Physics, MaiTai, tunable between 690 and 1040 nm, repetition rate = 84 MHz and pulse duration = 120 fs), (b) **fs2**: regenerative amplifier (Spectra-Physics, Spitfire, 800 nm, repetition rate = 1 KHz and pulse duration = 120 fs). For 1PA-PL excitation, the sources are either from 410 nm CW diode laser or frequency doubled 800 nm **fs1** and **fs2** lasers. For 2PA-PL measurements, the excitation is from 800 nm **fs2** laser and/or selective wavelengths from 780 to 1000 nm of **fs1** laser. Fiber optic coupled spectrometer is used to collect the spectra through appropriate high and low pass filters.

### Time resolved photoluminescence

Time resolved photoluminescence measurements on thin films were carried out in both Time Correlated Single Photon Counting (TCSPC) detector (upto 10 ns) as well as Up-Conversion module (upto 300 ps) utilizing **fs1** laser. For 1PA-PL dynamics the 400 nm pulsed excitation was provided by **fs1** laser by frequency doubling the fundamental 800 nm. For 2PA-PL, various wavelengths between 690 and 1040 nm from **fs1** laser are used. The generated PL was directly coupled to the TCSPC detector (SPC-130). In the case of upconversion module, the emitted PL after excitation at 400 nm was mixed with 800 nm laser producing upconverted signal. This signal was coupled to the photomultiplier tube attached with the monochromator.

### Imaging and microscopy

Simultaneous measurements of high resolution bright field image (BF), PL image and spectra, PL spatial spectral mapping of CHPI single crystal platelets were performed utilizing an optical upright microscope (BX51, Olympus). Three types light inputs (a) 410 nm CW laser, (b) femtosecond mode-locked oscillator laser (Spectra Physics, MaiTai, λ = 800 nm and 400 nm, 120 fs) and (c) conventional Xe/Hg lamps. The modified microscope output is coupled to CCD camera and fiber optic spectrometer for image and spectra respectively. Spatial mapping is carried out by a controlled motorized X-Y stage.

## Supplementary information

Supplementary information.
