# Supplementary material for: Linear and nonlinear optical probing of various excitons in 2D inorganic-organic hybrid structures
Source: Sci Rep. 2020 Feb 13;10:2615. doi: 10.1038/s41598-020-59457-7 (PMC7018830; doi:10.1038/s41598-020-59457-7)
Supplement: Supplementary file 1 — Supplementary information. [file 41598_2020_59457_MOESM1_ESM.docx]

**Supplementary Information**

**Linear and nonlinear optical probing of various excitons in 2D inorganic-organic hybrid structures**

Mohammad Adnan^1^, Jeremy J. Baumberg^2^ and G. Vijaya Prakash^1^*

*^1^Nanophotonics Lab, Department of Physics, Indian Institute of Technology Delhi,*

*New Delhi 110016 India*

*^2^Nanophotonics Centre, Cavendish Laboratory, University of Cambridge,*

*Cambridge, CB3 0HE, United Kingdom*

Figure S1 (a) shows excitation wavelength dependent 2PA-PL spectra from 780-1000nm at fixed excitation intensity 4GW/cm^2^ from **fs1** laser. Inset shows the plot of 2PA-PL peak position shift with the tuning of excitation wavelength (PLE). (b) shows the excitation intensity dependent 2PA-PL spectra, excited with 800nm fs1 laser. The inset shows the plot of 2PA-PL peak shift with the incident intensity. This is to be noted that cumulative local heating is typically possible in semiconductors due to high laser pulse repetition rate (84MHz). Such local heating may modify the [PbI_4_]^2-^ network which will be directly reflected in the bandgap and exciton energies^47-50^. However, these intensity dependent 2PA-PL spectral studies (Fig. S1b) shows only moderate red-shifting of peak position at a rate of 1.6nm per GW/cm^2^ excitation intensity.

Figure S2 (a) Excitation intensity dependent time-resolved 2PA-PL dynamics for CHPI film. Inset shows extracted lifetimes vs incident laser intensities. (b) shows the excitation wavelength dependent time-resolved 2PA-PL dynamics. Inset shows extracted lifetimes vs excitation wavelength. Excitation intensity was fixed at 3GW/cm^2^ from **fs1** laser

Figure S3 (a) shows 2PA-PL spectra at very high excitation (> 10^13^W/cm^2^) intensities utilizing 800nm **fs2** laser. (b) shows the peak PL intensity plot vs incident excitation intensities. At this excitation intensities (damage threshold region) the spectra experience considerable peak shifts and PL intensity degradation, likely due to the sample damage and/or structure deformation. See Figure S7 for laser exposure stability.

**
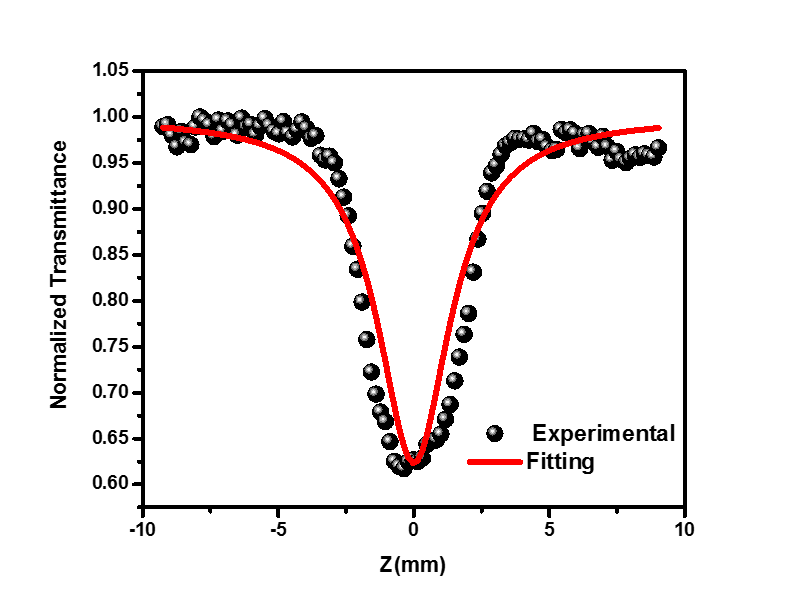
**

Figure S4 (a) Open aperture Z scan data for CHPI thin film, Fitting according equation^50^ $T_{norm}=\frac{ln[1+q_{0}(z)]}{q_{0}(z)}$ where $q_{0}=\frac{\beta I_{0}L_{eff}}{1+{(\frac{z}{z_{0}})}^{2}}$. Irradiation Intensity is 1.6x10^12^W/cm^2^ at 800nm (>75fs, 1KHz). Further experiments to establish the various nonlinearities involved in these IO hybrid are under progress.


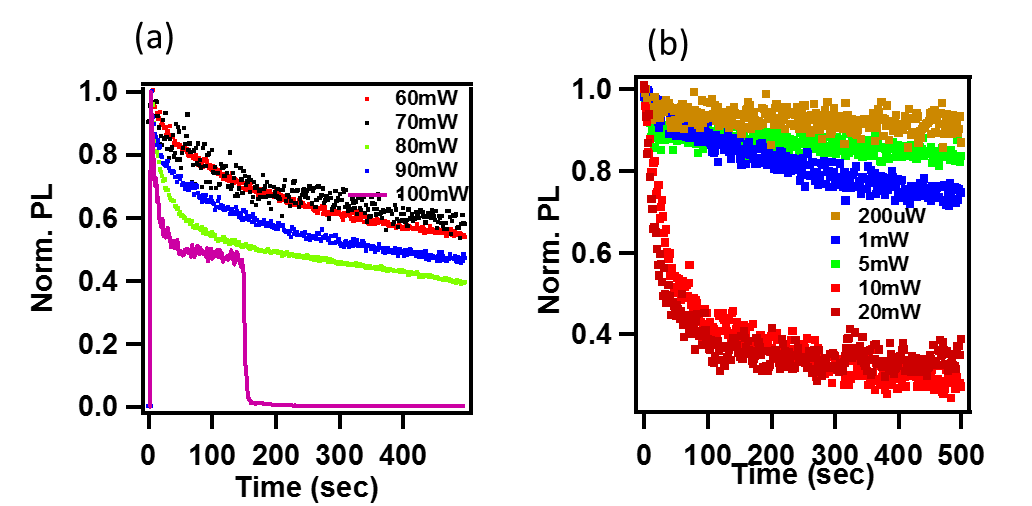


Figure S5. Laser irradiation stability: 2PA-PL peak (530nm) intensity vs continuous exposure time. Excitation sources (a) 800nm **fs1** and (b) 800nm **fs2** lasers. For **fs1** average powers ~100mW (~10^10^W/cm^2^) and for **fs2** average powers ~5mW (~3x10^13^W/cm^2^) are the usage limits, beyond which the sample degradation occurs.

Figure S6. Same as Figure 2(b&c), here we show the multi exponential fitting, showing various slopes (s). The slopes >1 is clearly represents various high fluence excitation dependent nonlinear phenomena related one-photon (saturation of absorption and exciton-exciton annihilation) and two-photon absorption processes.

Figure S7. Same as Fig 3(a), here we exemplify 1PA-PL life time fittings (black dark lines) for both longer time scales (from Time Correlated Single Photon Counting (TCSPC), range ~0-10ns, resolution ~80ps) and short time scales (Up-conversion mode detection, range 300ps, resolution ~1ps). This representative data is for the excitation intensity I=2.29GW/cm^2^ at 400nm fs1 laser. Longer time scaled TCPSC clearly shows bi-exponential behavior ( τ_1_ = 207ps and τ_2_ = 1083ps) and short time scales predominantly shows a single exponential ( τ_1_ = 36ps).

Figure S8 Modified optical microscope equipped with excitation sources 400nm CW, 800nm (400nm) fs1 lasers, Xe and Hg lamps. This modified microscope facilitates (a) dark and bright field images (b) 1PA- and 2PA-PL image, (c) spatial spectral mapping and (d) single point PL spectra.

Figure S9 (a,b) 1PA-PL and (c) 2PA-PL Optical microscopic PL images and Spatial PL intensity mappings of CHPI crystal platelet. Excitations are (a) 365nm (Hg lamp), (b) 400nm (**fs1** laser) and (c) 800nm (**fs1** laser) respectively.

Figure S10 (A) 1PA-PL (400nm, fs1 excitation) and 2PA-PL (800nm, fs1) spatial PL peak intensity mappings of CHPI single crystal platelet (same as Fig 4). (B) Spectral resolved horizontal line scan at the middle of the crystal. (C) Normalized 1PA and 2PA PL spectra extracted at various points on the single crystal platelet (from Fig B). Having irregular crystal surfaces, the PL spectra at various positions can be visualized as the PL collection from varied depths/thickness. The observed peak shift in both kinds of PL with respect to varied thickness/depth is only about 2-3nm, while the peak difference between 1PA and 2PA PL is as large as ~17nm.
